# Reducing Central Line-associated Bloodstream Infections Using a Frontline Staff-driven Approach

**DOI:** 10.1097/pq9.0000000000000685

**Published:** 2024-02-21

**Authors:** Joanne Pasinski, Johanna Young, Nicole Leone, Kaitlyn Philips

**Affiliations:** 1 From the Department of Pediatrics, Joseph M. Sanzari Children’s Hospital, Hackensack University Medical Center, Hackensack N.J.

## Background:

Infections are a major source of morbidity and mortality for infants in the neonatal ICU (NICU).^1^ Sustainable improvement in our central line-associated bloodstream infection (CLABSI) rate was challenging, despite adherence to evidence-based best practices.^2–4^ Therefore, we leveraged a multidisciplinary approach to reinvigorate our improvement efforts and reduce the CLABSI rate. We aimed to decrease the CLABSI rate from 2.97 to 1.49 infections (a 50% reduction) per 1000 central line days for infants of all birth weights over 36 months.

## Methods:

We used the Model for Improvement and plan, do, study, act cycles as the framework for our improvement effort.^5^ Stakeholders from frontline nursing, nursing leadership, physician staff, advanced practitioners, administrators, and families formed a multidisciplinary team. A frontline nurse led this team and held staff accountable. Interventions were multi-factorial, agreed upon by the stakeholder team, and included all staff members taking ownership of line maintenance. Videos demonstrating two-person sterile procedures, such as line and cap changes, standardized practice throughout the unit. Educational materials introduced the importance of central line maintenance and infection prevention to families. Daily verification of occlusive central line dressings by the bedside nurse and neonatologist encouraged multidisciplinary accountability for central line care. A newsletter was created to maintain communication about process changes and provide performance feedback to frontline staff. Aligning our practice with current parenteral nutrition guidelines led to increased frequency of tubing changes, from every 96 to every 24 hours. Staff incorporated new products, such as adhesives and dressings to reduce the number of dressing changes overall. The outcome measure was the CLABSI rate. The process measure was compliance with the CLABSI prevention bundle for line maintenance. These data were collected monthly and analyzed for special cause variation on a statistical process control chart (primary outcome) and run chart (process measure).

## Results:

**Fig. 1.**
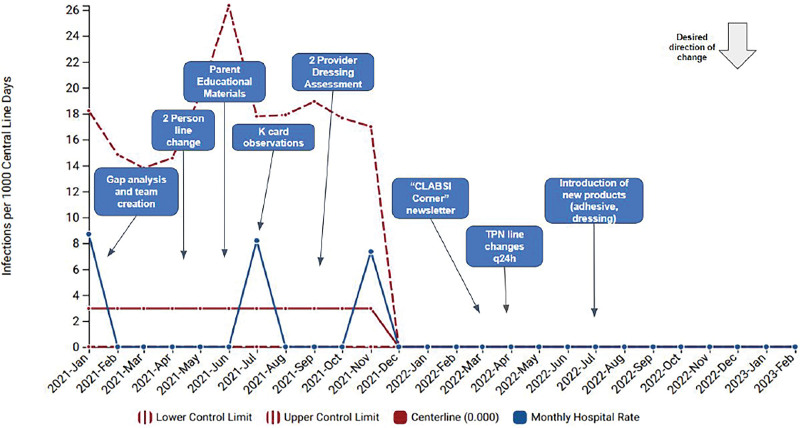
CLABSI rate in NICU. Includes both mucosal barrier injury (MBI) and non-MBI infections.

**Fig. 2.**
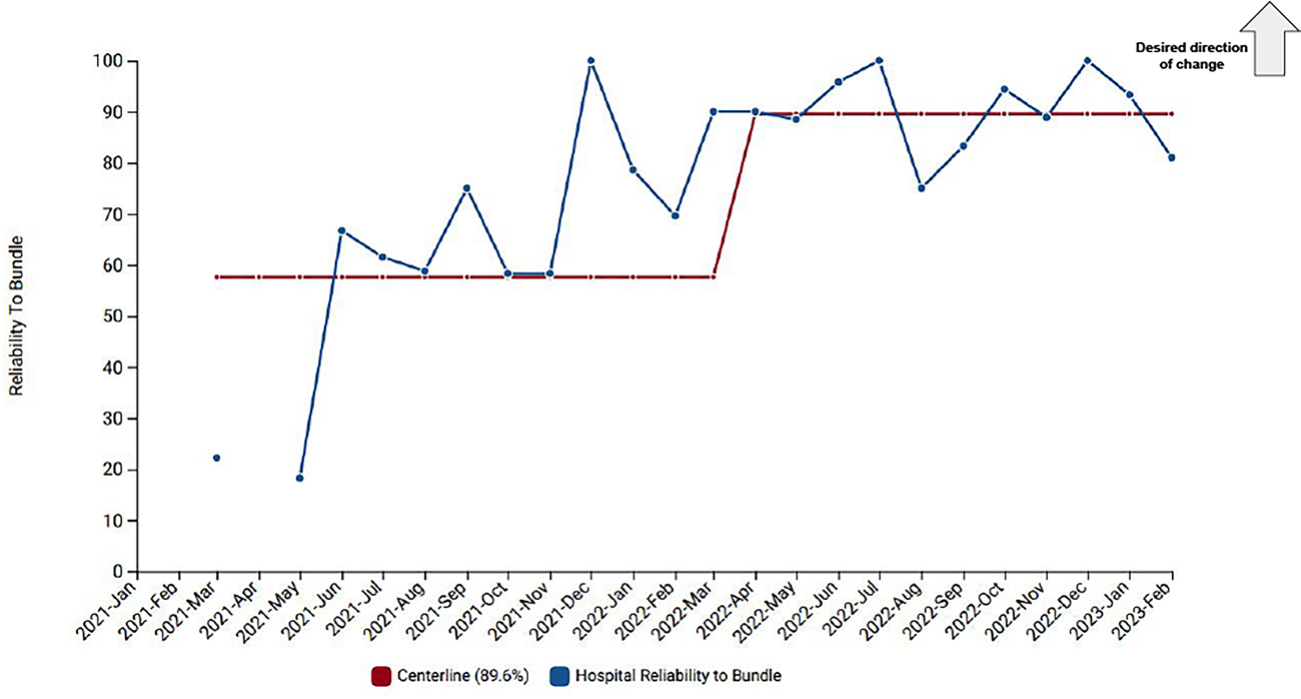
Run chart of reliability to CLABSI prevention bundle in NICU.

The baseline CLABSI rate in our unit was 2.97 infections per 1000 central line days. After multiple plan, do, study, act cycles, we demonstrated special cause variation and a shift of the centerline to zero (Fig. 1). The unit sustained a CLABSI rate of zero for over 500 days. Prevention bundle reliability improved from 57.6% to 89.6% (Fig. 2). A frontline staff-driven approach and multidisciplinary team accountability was instrumental in creating this culture change.

## Conclusions:

Through a combination of frontline staff leadership, reinforcement of best practices, team accountability for central line maintenance, and adoption of new innovations, our team reduced our CLABSI rate to zero and sustained a CLABSI-free NICU for over 500 days.
